# Molecular characteristics of Hepatitis B and chronic liver disease in a cohort of HB carriers from Bamako, Mali

**DOI:** 10.1186/s12879-015-0916-x

**Published:** 2015-04-11

**Authors:** Fatou Traoré, Emmanuelle Gormally, Stéphanie Villar, Marlin D Friesen, John D Groopman, Guy Vernet, Souleymane Diallo, Pierre Hainaut, Moussa Y Maiga

**Affiliations:** Centre d’Infectiologie Charles Mérieux, Bamako, République du Mali; Université de Lyon, UMRS 449 ; Laboratoire de Biologie générale, Université Catholique de Lyon ; Reproduction et développement comparé, EPHE, Lyon, France; International Agency for Research on Cancer, Lyon, France; Bloomberg School of Public Health, Johns Hopkins University, Baltimore, USA; Laboratoire des Pathogènes Emergents Fondation Mérieux, Lyon, France; Centre Pasteur du Cameroun, Yaoundé, Cameroun; International Prevention Research Institute, Lyon, France; Service de Gastroentérologie et Hépatologie, Centre Hospitalier Universitaire Gabriel Touré, Bamako, République du Mali

**Keywords:** HBV, Viral load, Chronic carriage, Fibrotest/Actitest, *TP53 R249S* mutation, Mali

## Abstract

**Background:**

Hepatitis B (HB) infection is common in Mali. However, there is little information on molecular and biochemical characteristics of HB carriers.

**Methods:**

A group of 1466 adult volunteers was recruited in the district of Bamako. Confirmed HB carriers were tested for HB viral load by quantitative PCR and HBV was genotyped by sequencing of *HBS*. Fibrosis and hepatitis activity were measured using the Fibrotest-Actitest. A mutation of *TP53* at codon 249 (*R249S*), specific for exposure to aflatoxin, was detected in cell-free DNA extracted from plasma.

**Results:**

Overall, 276 subjects were HBsAg-positive (18.8%). Among 152 subjects tested for HBV load, 49 (32.2%) had over 10^4^ copies/mL and 16 (10.5%) had levels below the limit of detection. The E genotype was found in 91.1% of carriers. Fibrotest scores ≥ F2 were observed in 52 subjects (35.4%). Actitest scores ≥ A2 were detected in 15 subjects (10.2%) and were correlated with Fibrotest scores (p = 0.0006). Among 105 subjects tested, 60% had detectable levels of *R249S* copies (>40 copies/mL plasma).

**Conclusion:**

Chronic HB carriage in adults in Bamako district is well over epidemic threshold. About 1/3 of carriers have moderate to severe liver fibrosis and 60% have detectable aflatoxin-related *TP53 R249S* mutation. These results support introduction of anti-HB therapies to reduce the progression towards severe liver disease.

## Background

Chronic carriage of Hepatitis B Virus (HBV) reaches endemic levels (≥8% of the general populations) in many parts of Sub-Saharan Africa, South East Asia and Latin America. HB chronic infection is the main attributable risk factor worldwide for severe chronic liver disease and, significantly, hepatocellular carcinoma (HCC) [1,2]. In West Africa, where rates of chronic HB carriage in the general, non-vaccinated populations are 10-18%, HCC is the leading cancer in males (standardized incidence rates (ASRW) of 15-30/10^5^ person years) and the third in females (ASRW of 7-15/10^5^ person years) [3]. Most HCC patients are aged 35–50 and present with advanced tumors with an average time since first symptoms of only 2–3 months. Very few patients are diagnosed with cirrhosis before HCC detection. A clinical study in The Gambia has shown that, at HCC diagnosis, ultrasonography reveals concomitant cirrhosis in about 65% of the cases, corresponding in many cases to reactive, rather than precursor cirrhosis [4].

In West Africa, HBV infection mostly occurs perinatally through horizontal or vertical transmission and the rate of carriage by 15 years in non-HB vaccinated subjects is almost equal to adult rates [5,6]. In The Gambia, HB carriage is detected in over 90% of patients with HCC before age 50 [7]. Alcohol is not a significant risk factor at population level. In association with HBV, the other main risk factor for HCC is dietary exposure to aflatoxin-B1 (AFB1), a mycotoxins produced by the fungus *Aspergilus sp*. which contaminates several staple foods in semi-arid and tropical regions. Exposure to AFB1 induces a “signature” mutation in the tumour suppressor gene *TP53* (*R249S*, AGG to AGT at codon 249, arginine to serine substitution). This mutation has been detected in about 50% of HCC cases in The Gambia [8]. Using a sensitive and quantitative mass spectrometry method, Short-Oligonucleotide Fragment Analysis (SOMA), *R249S* is also detectable as a marker of current exposure in subjects exposed to AFB1 in the general population [9].

In Mali, studies in specific population groups (pregnant women, students, blood donors) reported a variable rate of HB carriage in adults ranging between 10 and 18% [10-17]. In a cohort of 91 patients receiving treatment for chronic hepatic diseases in hospital Gabriel Touré (Bamako), 54% were found to be HB carriers, compared to 4.3% in a matched group of blood donors used as controls [18]. In the same center a year-long longitudinal study of 57 patients with severe chronic liver disease, jaundice and ascites were observed to be the main clinical signs. In these patients mortality over 1 year was 82.5%, with the three main causes being HCC, abdominal bleeding and hepatic encephalopathy [19]. In this study, we have analyzed serological and molecular parameters of HB carriage in a group of 152 adults unselected for pathological conditions and recruited in outpatients clinics and in several occupational groups. The 152 participants were confirmed positive for HBsAg at Center for infectiology Charles Merieux (CICM) in Bamako, Mali. Carriers were tested for fibrosis status and hepatitis activity by Fibrotest-Actitest and the presence of mutated *TP53* R249S DNA, a marker of mutagenesis by aflatoxin, in the serum or plasma.

## Methods

### Population and recruitment of subjects

**Figure 1:**
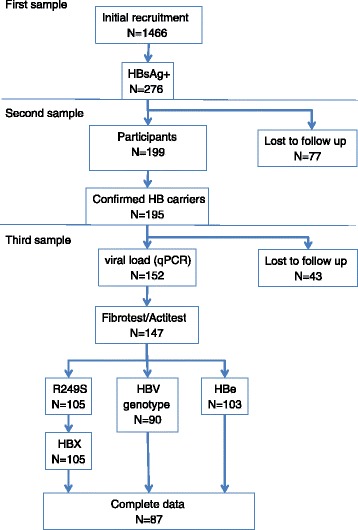
Flow chart of subject recruitment and selection for analysis. 1466 subjects were initially recruited. Due to practical reasons in a resource-constrained context (in which extensive and systematic follow-up of healthy subjects is not feasible), studies on biomarkers have been carried out on second or third blood samples collected at recall of subjects who were confirmed chronic carriers. From the initial recruitment, a total of 199 subjects reported for the first recall and carrier status was confirmed for 195 subjects. Among these subjects, 152 reported for a third sample, which was used to measure viral load (152 subjects) and to perform Actitest/Fibrotest (147 subjects; data on 5 subjects not available due to experimental failure). Among these 147 subjects, DNA could be extracted from the plasma for 105 subjects, who were tested for R249S, HBX (n = 105), HBe testing (n = 103) or HBV genotype (n = 90). The reduction in numbers for HBe and HBV genotype is due to experimental failure.

A total of 1466 adult volunteers were recruited between March and September 2009. These volunteers were contacted through on-site information among different groups, including students (Faculty of Medicine and Pharmacy and National School for Police, Bamako), university hospital staff and visitors (Kati hospital; Point G and Gabriel Touré hospitals in Bamako) (Figure 1). None of the subjects recruited had been referred to this study on the basis of prevalent hepatic symptoms and none had been previously tested for HB status. At the start of the study, a blood sample (4 mL) was obtained by venipuncture using standard vacutainers. Blood samples were processed immediately for initial serological tests. A second sample was obtained at least 6 months after the initial sample. This second sample was used for confirming the HB carrier status. Confirmed carriers were requested to complete a questionnaire with basic demographic information, history of liver disease and hepatitis. A third sample was obtained from 152 subjects and was used for biochemical and molecular tests. Molecular analyses were performed using blood products (plasma) stored at −80°C. Prior to inclusion into the study, subjects received individual information by a medical doctor of the Hepato-Gastroenterology department, Hospital Gabriel Touré and oral informed consent was obtained. The study protocol was approved by the ethical committee of INRSP (Institut National de Recherche en Santé Publique, decision N°09-000008/CE-INRSP, 19^th^ of May 2009), according to national regulations in Mali.

### Serology

All subjects were screened for HBsAg using One-Step HBsAg test (SD BIOLINE Hepatitis test) and subjects “positive” with this test were confirmed on an independent blood sample using VIDAS HBs Ultra (bioMérieux). Among “negative” subjects with One-Step HBsAg test, 10% were tested using VIDAS HBs Ultra and were all confirmed negative. The confirmed HB carrier status was established by a second, independent test performed on a blood sample obtained 6 months after the initial analysis. HBe was detected on a subset of 98 HBsAg + subjects who presented for a second sample, using ELISA (VIDAS, bioMérieux). HCV was similarly detected using One-Step HCV test (SD BIOLINE Hepatitis test).

### Biochemistry

Confirmed HB carriers who presented for a third blood sample (at least 6 months after initial testing) were evaluated for the status of liver fibrosis and necrotic-inflammatory hepatitis B using Fibrotest/Actitest [20,21]. These tests were performed at Biomnis (France). Results were expressed as Fibrosis (F0-F4) and Hepatitis activity (A0-A3), matched to METAVIR scores as described by the manufacturer [20].

### Viral load and HBV genotyping

HB viral load was measured by quantitative PCR (qPCR) using the COBA AmpliPrep/COBAS TaqMan HBV Test v2.0 (Roche) as described by the manufacturers using 4 mL of frozen plasma. Viral load was expressed in number of HBV copies/mL of plasma. HBV genotyping was performed by sequencing the *HBS* gene in circulating-free DNA extracted from plasma (300 μl) using the QIAmp DNA Blood Mini Kit (Qiagen), as previously described (Villar et al., [9]). A semi-nested PCR was used to amplify the entire S gene [22]. The first reaction used 5 μL DNA with primers S_HBVpol1 (5′-cctgctggtggctccagttca-3′) and S_HBVporv2 (5′-aaagcccaaaagacccacaat-3′). The PCR reactions were performed as described before [22]. The second step used 1 μL first reaction product and primers S_HBV123s (5′tcgaggattggggaccctg-3′) and S_HBVporv2. The PCR reactions were performed as described before [22]. PCR products (5 μL) were purified using standard ExoSap-IT enzyme, (USB Corporation) and nucleotide sequences were determined for both strands by automated dideoxy sequencing (AbiPrism 3100 sequencer; Applied Biosystems). Direct sequencing on amplified fragments used the primers S_HBV123s, S_HBVporv2, and S_HBV778r (5′-gaggtataaagggactcaag-3′). Alignment of *HBS* sequences on HB sequences from database sequences were determined using genotyping algorithms and tools available at HBVRegDB (http://lancelot.otago.ac.nz/HBVRegDB/index.php?page=Tutorials).

### Detection of TP53 R249S

Aliquots of the same DNA extracts used for *HBS* sequencing were used to measure mutant *TP53* R249S plasma concentrations by quantitative SOMA using an internal standard plasmid [9,23]. Briefly, 228 copies of an internal standard plasmid were added to all DNA extracts to provide a reference for quantitation before PCR amplification of a 93 base-pair segment of exon 7 of *TP53* encompassing codon 249. After restriction digestion with *Hae*III, which specifically cuts only the wild-type sequence of codon 249, the mutated and internal-standard–enriched PCR products were re-amplified and cut with *Gsu*I to produce 8-mer oligonucleotides. The latter were purified and quantitated by HPLC/electrospray ionization tandem mass spectrometry. Results were expressed as concentrations (copies of *R249S*/mL plasma). Plasma levels of *R249S* DNA ranged from non-detectable to 63,800 copies/mL. Samples with plasma concentrations > 40 copies/mL were classified as positive for *R249S*.

### Analysis of HBX gene status in plasma DNA

Detection of truncated versus complete integrated *HBX* sequences was performed by PCR as previously described [24] and modified methods [25,26]. DNA was amplified by PCR to produce four overlapping amplicons from *HBX* of 139, 192, 334 or 425 bp. The 425 bp amplicon encompasses the entire *HBX* sequence and the three shorter amplicons correspond to fragments initiated at the 5′-end of *HBX* and covering progressive lengths of its sequence. Amplification of all four fragments signals the presence of a complete *HBX*, whereas amplification of one or several shorter fragments signals the presence of 3′-truncated *HBX*. The same forward primer X1F was used in each reaction (5′-GGGACGTCCTTTGTCTACGT-3′). The four reverse primers were X1R (5′-GGGAGACCGCGTAAAGAGAG-3′), X2R (5′-GTGCAGAGGTGAAGCGAAGT-3′), X3R (5′-CCCAACTCCTCCCAGTCTTT-3′) or X4R (5′-GGCAGAGGTGAAAAAGTTGCA-3′). PCR conditions have been described previously [27]. The identity of all PCR products was confirmed by a second PCR and sequencing.

### Statistical analysis

Univariate Chi-square tests and p-values, Fisher-Exact tests and Odd Ratio (OR) calculations were carried out using the statistical analysis tools available at http://vassarstats.net/. A p value ≤0.05 was considered as significant.

## Results

### Characteristics of HB carriers

Figure 1 summarizes the recruitment of subjects and the number of subjects tested for the various biomarkers measured. Of the 1466 subjects who volunteered for this study, a total of 276 (18.8%) were identified as HBsAg + using a rapid screening test which was subsequently confirmed using a standard reference test (VIDAS, bioMérieux). Of these 276 subjects, a total of 199 provided an additional blood sample at least 6 months after the initial screening sample and 195 of them were re-confirmed to be HBsAg+. These subjects are therefore identified “confirmed HB carrier”. The recruitment and demographic characteristics of these subjects are listed in Table 1. The average age was 35.1 ± 11.1 (range: 18–69 years) and the gender ratio (M/F) 2.68:1. Of these subjects, 110 (52.4%) were recruited among persons attending hospital outpatient clinics (including mostly patient’s family members and visitors) at Hospital Gabriel Touré and Point G, Bamako. The other recruited subjects were members of several specific groups (military staff, medical students and health staff). The majority of the subjects were civil servant (118; 60.5%), followed by students (45; 23.1%). HB carriage in the different recruitment groups was 16.8% among military staff, 17% among medical students and health staff, and 23% among hospital attendants (P = 0.021, Chi-square Test).

**Table 1:** **Characteristics of confirmed HB carriers (n = 195)**

**Parameters**	**Number of participants**	**Percentage**
**Age groups (in years)**		
18-25	47	24.1
26-35	66	33.9
36-45	46	23.6
46-55	25	12.8
≥56	11	5.6
**Sex**		
Male	142	72.8
Female	53	27.2
**Recruitment sites**		
Visitors, Hospital Gabriel Touré*	93	47.7
Visitors, Hospital Point “G”*	17	8.7
National Police School/military staff	53	27.2
Medical staff, Hospital Gabriel Touré	17	8.7
Medical students	15	7.7
	195	
**Occupation**		
Civil servants	118	60.5
Students	45	23.1
Housewives	14	7.2
Traders	7	3.6
Primary school students	4	2.1
Workers	3	1.5
Others	4	2.1

*Recruited at hospital’s general admission.

Among the 195 HB carriers, a group of 152 subjects reported for a third sample, which was used for quantitation of viral load in the plasma using qPCR. The average viral load was 3.2 ± 1.7 log_10 ._ HBV DNA was undetectable in 16/152 subjects (10.5%), whereas a load >10^4^ copies/mL plasma was detected in 49/152 subjects (32.2%) (Table 2). Among these 152 subjects, HBeAg status was available for 98 subjects. A total of 7 subjects were tested positive for HBe (7.1%). Of note, these 7 subjects had a viral load ≥ 10^7^ copies/mL, underlining the strong correlation between viral load and HBe + status. HCV was detected using a rapid screening test in 44 of the initial group of 1466 volunteers (3%) (data not shown). Among the 152 HB carriers who were assessed for molecular markers of HB infection, only one was also HCV-positive.

**Table 2:** **HBe status (n = 103), HBV viral load (n = 152) and TP53 R249S mutant DNA plasma concentration in confirmed HB carriers**

**Parameter**	**Number of participants**	**Percentage**
**HBe Antigen**		
Positive	7	6.8
Negative	96	93.2
**Total**	103	100
**Viral load (copies/mL)**		
Non-detectable (Log 1)	16	10.5
10 -10,000 (Log1 - < log4)	87	57.2
>10,000 (Log4 - Log8)	49	32.2
**Total**	152	100
**TP53 R259S mutant DNA (copies/mL)**		
0-40	42	40
41-100	22	21
101-500	29	27,6
501-1000	10	9,5
1001-2000	2	1,9
**Total**	105	100

### Genotyping and analysis of HBX status

Complete *HBS* sequences were generated for 90 subjects. A total of 82 (91.1%) were classified as HBV genotype E. A further 5 subjects (5.6%) showed sequences which matched both E and D genotypes. Three subjects were found to carry either D4 (1 subject) or A3 (2 subjects) genotypes. There was no difference between age and biochemical parameters of liver disease according to different genotypes. *HBX* gene status (complete sequence or 3′ truncated) was analyzed by PCR in 105 subjects. Only 6 subjects (5.7%) had undetectable *HBX* sequences. Complete sequences were detected in 60 (57.1%) and truncated sequences in 39 (37.1%) subjects.

### Biochemical characterization of hepatitis and liver disease

Fibrotest-Actitest was performed in 147 subjects (Table 3). Results were expressed as conversion in METAVIR scores (F0-F4 for Fibrotest and A0-A3 for Actitest). For Fibrotest, Fibrosis scores from F0 to F1 were considered as “minimal or absent fibrosis”, F1-F2 to F2 as “moderate fibrosis” and F3 to F4 as “severe fibrosis”. Whereas a total of 95 subjects (64.6%) were scored as having minimal or absent fibrosis, 33 subjects (22.5%) were scored as having moderate and 19 (12.9%) severe fibrosis. Thus, a total of 35.4% had significant biochemical signs of fibrosis. There was no correlation between the distribution of these three score groups and viral load (p = 0.801, Chi square test). For Actitest, viral necrotic-inflammatory activity scores A0 –A1 were considered as “minimal or absent activity”, A2 as “moderate activity” and A3 as “high activity”. 132 subjects (89.8%) were scored as having minimal or absent activity, 10 (6.8%) as having moderate activity and 5 (3.4%) as having high activity. There was no statistically significant association between Actitest scores (grouped into two categories, minimal/absent *vs* moderate/high) and viral load (either <10^4^ or ≥ 10^4^, p = 0.18, Fisher Exact test). In contrast, there was a strong correlation between Fibrotest and Actitest scores (each grouped as dichotomized variable, minimal/absent *vs* moderate/severe or high). The OR for having F scores ≥ 2 was 9.2 (95% CI [2.5-34.4]) in subjects with A scores ≥ 1, as compared with subjects with A and F scores falling in the “minimal/absent” category (P = 0.000267, Fisher Exact test). On the other hand, F scores were associated with *HBX* gene status. OR for having F scores ≥ 2 was 4 (95% CI [1.6-9.9]) in subjects with complete *HBX* sequences as compared to subjects with truncated sequences (P = 0.0019, Fisher Exact test). In contrast, there was no significant association between A scores ≥2 and *HBX* status.

**Table 3:** **Conversion to METAVIR score of Fibrotest-Actitest in 147 confirmed HB carriers**

**METAVIR score**	**Number of participants**	**Percentage**
**Fibrosis**		
F0	69	46.9
F0-F1	20	13.6
F1	6	4.1
F1-F2	23	15.7
F2	10	6.8
F3	11	7.5
F4	8	5.4
**Total**	147	100.0
**Necrotic-inflammatory activity**		
A0	84	57.1
A0-A1	38	25.9
A1	10	6.8
A1-A2	5	3.4
A2	5	3.4
A3	5	3.4
**Total**	147	100.00

### Detection of R249S and HBX gene status in plasma DNA

The distribution of mutant *TP53* R249S DNA concentrations detected by qSOMA in free circulating DNA from the plasma are shown in Table 2. Among 105 subjects tested, 63 (60%) had detectable R249S in the plasma (≥40 copies/mL). The mean number of copies in subjects with ≥40 copies/mL was 311 (SD: 340) copies/mL, with a median of 162 copies/mL (min: 42; max 1784). There was a borderline tendency for association between low/undetectable R249S and F scores ≥2. Compared to subjects with ≥40 R249S copies/mL, subjects with ≤40 copies/mL had an OR of 2.1 (95% CI [0.9-4.8]) of having moderate to severe fibrosis (P = 0.06; Fisher Exact test). There was no statistically significant association between positivity for R249S and *HBX* gene status (complete or truncated).

## Discussion

We have studied a group of volunteers recruited from various groups of the population in the district of Bamako, Mali, for HB carriage (as defined by persistent HBsAg + status) and we have further characterized several molecular, serological and biochemical parameters in the sub-set of those who were confirmed HB carriers. The results show that the rate of HB chronic carriage is high in this study population (18.8%), and that at least 1/3 of the carriers have significant signs of liver fibrosis (moderate to severe Fibrotest scores, ≥F2, 35.4%) or active hepatitis (viral load ≥ 10^4^ copies/mL, 34.2%). Thus, a substantial proportion of these HB carriers with clear signs of active hepatitis might benefit from anti HB-treatment for reducing the risk of progression towards severe liver disease or liver cancer, in particular in a context where HB carriers have detectable mutagenic exposure to aflatoxin [28-30].

The population recruited for this study includes volunteer hospital visitors and members of several occupational groups, unselected for pathology or condition. The focus on such defined groups may cause a selection bias. It should be noted that previous studies in Mali have been conducted on particular groups of high-risk (e.g. patients with liver disease, HIV-positive subjects, [11,18,19]) or low-risk (e.g. blood donors, [10,12,17]) subjects. Several studies have analyzed seroprevalence in pregnant women and in women of childbearing age [13-16]. Studies in blood donors and in healthy women reported a prevalence of HB carriage ranging between 10 and 18%. The proportion of carriers in the present study group (18.8%) is therefore in the high range of these previous estimates and is also among the highest reported in studies in West Africa. Rates between 11 and 17% have been consistently observed in studies performed in The Gambia or in Senegal [4,7,31]. However, most of these studies have been carried out on selected “control” groups and data from The Gambia are for an essentially rural population [4,7].

Of note, childhood HB vaccination has been introduced in the Expanded Program of Immunization (EPI) in Mali in 2002. It is expected that, provided adequate vaccination coverage and efficacy, the rate of HB carriage in the general population will decrease in the next two-three decades [32].

The volunteers recruited in this study were categorized in different sub-groups according to their social/professional status and the context of their recruitment. Rates of HB carriage of 16.8% and 17% were detected in two professional groups, respectively, military staff and staff recruited in a hospital setting (students; nurses and other hospital staff). In contrast, high rates (23%) were observed in non-patient volunteers recruited at outpatient clinics in major hospitals in Bamako city (Hospital Gabriel Touré and Point G), and the difference between the three recruitment groups was found to be significant (P = 0.0021). The social, demographic and health status of subjects in these “non-patient” subjects recruited in a hospital setting is not precisely known. We have noted that this group is composed of a mixed population with generally lower socio-economic status than the two professional groups. Therefore, further studies on the rate of HB carriage in different strata of the Malian population are needed. It is indeed possible that the rate of carriage might be even higher than the average reported here in less-economically favored groups of the population.

The structure of the study was such that subjects who were tested as HBsAg + at initial recruitment (276 positive subjects) were recalled for subsequent blood samples for other analyses at different time point in the study. Only part of the initial HBsAg + group was available for these subsequent analyses, which were thus carried out among subjects from a smaller series of 195 confirmed HB carriers (Figure 1). Thus, we cannot rule out a selection bias due to compliance with recall. However, there is no indication that a particular recruitment sub-group was more represented in the recall group than in the initial recruitment population.

Based on alignment of *HBS* sequences with reference database sequences, the most common HBV genotype in HB carriers was genotype E, present in 91.1% subjects. A small number of subjects were carriers of either D or A genotypes, and 5 subjects had sequences that matched both genotype E and D. The dominance of genotype E in this population is in agreement with studies in other West African populations. The only subject with A genotype had the A3 variant, a sub-genotype documented in West African populations [33]. The presence of both genotype E and D sequences in about 5.6% of the subjects is suggestive of either co-infection or recombination between these genotypes, an event that has been shown in a small number of infected subjects in different populations of West Africa [33].

Monitoring of HBV viral load is the most widely used method in assessing liver disease severity, predicting cirrhosis and HCC, deciding initiation of antiviral therapy, assessing treatment response and detecting the emergence of drug resistance. In this cohort, 89.5% of HBsAg + subjects (136/152) had detectable HBV viral load, with an average at 3.2 ± 1.7 log_10_ IU/mL and 32.2% (49/152) had loads over 4 log_10_ IU/mL, considered as cutoff value for high viral loads. Although there was an overall high correlation between HBe + status and viral load, over 75% of the subjects with viral load ≥4.0 log_10_ UI/mL were negative for HBe, an observation compatible with previous studies on HB carriers in The Gambia [34]. No previous data is available for chronic HB carriers in Mali. In Senegal, a study on 225 HB carriers recruited in a hospital setting in Senegal (Dakar) has detected an average viral load of 3.6 log_10_ IU/mL and 22% had viral loads ≥4.2 log_10_ UI/mL [35].

The use of Fibrotest and Actitest as surrogate endpoint to assess inactive HB carrier status and to predict liver disease-free survival has been evaluated in a cohort of 1,074 patients that includes 47% of patients of African origin [20]. This study showed that subjects with F scores ≤ 2.9 (corresponding to ≤ F1 status) and A scores ≤ 2.7 (corresponding to ≤ A1) had 100% of HB-complication-free survival after 4 years of follow-up, and that F scores outperformed combined classical predictors such as HBe antibody status, low AST activity, low viral load and absence of co-infections with HIV, HCV or HDV. Thus, normal Fibrotest and Actitest values have been proposed as criteria for a new definition of inactive HB carrier status. In the present study, of 100 subjects with complete data for viral load, Fibrotest and Actitest, 35 (35%) matched these criteria of inactive carrier status. Of these subjects, 8 (22.9%) had a viral load ≥ 4 log_10_ IU/ml. On the other hand, of 147 subjects tested by Fibrotest, 33 and 19 were scored as having moderate or severe fibrosis, respectively, representing together over 1/3 of the study participants (35.4%). There was no significant correlation between F scores and viral load. Regarding Actitest, 15 subjects had A status ≥ 2 (10.2%) and this score was highly correlated with a viral load ≥ 4 log_10_ IU/mL. Overall, 44% of the total number of subjects tested for viral load, Fibrotest or Actitest was scored as “positive” for at least one of the three tests. These observations suggest that a substantial proportion of HB carriers in the population may benefit from treatment with nucleoside analogs such as Adefovir or Tenofovir [36]. However, there is controversy on whether Fibrotest may represent an accurate method for the diagnosis of fibrosis in West-African HB carriers. A study in Dakar on HB carriers with normal AST levels and high viral load, found discordant results for the prediction of fibrosis using Fibrotest, Fibrometer (another biochemical test) or Fibroscan (liver stiffness measurements) [35]. It should be noted that markers such as Haptoglobin, included in both Fibrotest and Actitest, are strongly expressed in subjects infected by chronic malaria and sickle cell anaemia [37], two prevalent conditions in Bamako. Therefore, further studies taking into account the specific epidemiological context are needed to determine an appropriate algorithm for decision of initiating anti-viral treatment in West African populations.

Levels of *TP53* mutation at codon 249 (R249S) in circulating-free DNA from plasma have been proposed as a direct biomarker for the mutagenic effect of aflatoxin in normal subjects exposed through the diet. This mutation is present at high levels (>2500 copies/mL plasma) in liver cancer patients and its presence at such levels is concordant with mutation status in HCC tissues [38]. However, in asymptomatic subjects from The Gambia, low levels of R249S (70–2500 copies/mL) have been reported in subjects from the general rural population, with seasonal variations that may reflect variations in dietary intake of contaminated food [22]. In the present study, we have observed that about 60% of HB carriers from Bamako had R249S levels above threshold values (40 copies/mL), a proportion that is similar to HB carriers in rural Gambia. However, in subjects from Bamako, plasma concentrations (311 ± 340) were lower than in carriers from rural Gambia, in whom mean R249S levels detected at different periods of the year varied between 480 and 5,690 copies/mL serum. Overall, these results suggest that carriers form Bamako may have less exposure to aflatoxin than carriers from rural Gambia. This difference may reflect the combined effect of differences in dietary patterns in urban Bamako and rural Gambia, as well as the effect of interventions aimed at reducing crop contamination by aflatoxin, which are taking place in Mali since the mid 1990’s.

## Conclusion

In conclusion, this study provides a detailed assessment of HB chronic infection status in a group of subjects, unselected for clinical symptoms, in Bamako-Mali. Previous studies in Mali and in West Africa have mostly addressed HB status in specific groups such as blood donors [10,12,17], pregnant women [13,16], or patients diagnosed with chronic active hepatitis [35]. The results show that the rate of HB carriage in the population of Bamako is in the higher range of estimates for West Africa, and that about 30% of adult carriers have HB and/or liver biomarkers that would qualify them for treatment with HB anti-viral therapies. On the other hand, mean levels of mutant R249S DNA, a marker of exposure to aflatoxin, are significantly lower than those measured in rural Gambia, where aflatoxin-contaminated crops are an important component of the diet. Future studies are needed to assess importance of these factors for the risk of fatal liver disease and liver cancer in the general population of Bamako/Mali, in a context of epidemiological transition.
